# National priority setting partnership using a Delphi consensus process to develop neonatal research questions suitable for practice-changing randomised trials in the UK

**DOI:** 10.1136/archdischild-2023-325504

**Published:** 2023-04-24

**Authors:** Katie Evans, Cheryl Battersby, James P Boardman, Elaine Boyle, Will Carroll, Kate Dinwiddy, Jon Dorling, Katie Gallagher, Pollyanna Hardy, Emma Johnston, Helen Mactier, Claire Marcroft, James William Harrison Webbe, Chris Gale

**Affiliations:** 1 School of Public Health, Faculty of Medicine, Neonatal Medicine, Imperial College London, London, UK; 2 Neonatal Medicine, The University of Edinburgh MRC Centre for Reproductive Health, Edinburgh, UK; 3 Neonatal Medicine, University of Leicester, Leicester, UK; 4 Neonatal Clinical Studies Group, National Institute for Health and Care Research, London, UK; 5 Child Health, Keele University, Keele, UK; 6 British Association of Perinatal Medicine, London, UK; 7 Department of Neonatal Medicine, University Hospital Southampton NHS Foundation Trust, Southampton, UK; 8 EGA Institute for Women's Health, University College London, London, UK; 9 Policy Research Unit in Maternal Health & Care, National Perinatal Epidemiology Unit Clinical Trials Unit, University of Oxford, Oxford, UK; 10 Parents and Family Engagement Lead, Thames Valley and Wessex Operational Delivery Network, Thames Valley and Wessex, UK; 11 Neonatal Medicine, University of Glasgow, Glasgow, UK; 12 Neonatal Physiotherapy, Newcastle upon Tyne Hospitals NHS Foundation Trust, Newcastle upon Tyne, UK

**Keywords:** neonatology, health services research

## Abstract

**Background:**

The provision of neonatal care is variable and commonly lacks adequate evidence base; strategic development of methodologically robust clinical trials is needed to improve outcomes and maximise research resources. Historically, neonatal research topics have been selected by researchers; prioritisation processes involving wider stakeholder groups have generally identified research themes rather than specific questions amenable to interventional trials.

**Objective:**

To involve stakeholders including parents, healthcare professionals and researchers to identify and prioritise research questions suitable for answering in neonatal interventional trials in the UK.

**Design:**

Research questions were submitted by stakeholders in population, intervention, comparison, outcome format through an online platform. Questions were reviewed by a representative steering group; duplicates and previously answered questions were removed. Eligible questions were entered into a three-round online Delphi survey for prioritisation by all stakeholder groups.

**Participants:**

One hundred and eight respondents submitted research questions for consideration; 144 participants completed round one of the Delphi survey, 106 completed all three rounds.

**Results:**

Two hundred and sixty-five research questions were submitted and after steering group review, 186 entered into the Delphi survey. The top five ranked research questions related to breast milk fortification, intact cord resuscitation, timing of surgical intervention in necrotising enterocolitis, therapeutic hypothermia for mild hypoxic ischaemic encephalopathy and non-invasive respiratory support.

**Conclusions:**

We have identified and prioritised research questions suitable for practice-changing interventional trials in neonatal medicine in the UK at the present time. Trials targeting these uncertainties have potential to reduce research waste and improve neonatal care.

**WHAT IS ALREADY KNOWN ON THIS TOPIC**

- There is wide variability in neonatal care across the UK.
- Robust, high-quality interventional trials are the optimal approach to improving the evidence base and reducing variability in neonatal care.
- It is important to involve parents and other stakeholders in identifying important future research topics but this can be challenging and alternate approaches need to be developed.

**WHAT THIS STUDY ADDS**

- Previous prioritisation processes have identified broad themes of interest; this study identifies specific research questions suitable for answering in interventional trials.

**HOW THIS STUDY MIGHT AFFECT RESEARCH, PRACTICE OR POLICY**

- This prioritised list of specific research questions can be used by research organisations to support and develop practice-changing interventional trials within neonatology.

## Introduction

Neonatal clinical care varies widely,^1^ in part due to an incomplete evidence base for many treatments and approaches.^2^ The optimal way to resolve uncertainties in healthcare is through well-designed randomised controlled trials (RCTs).^3^ Such interventional studies require structured research questions that describe the participants, intervention(s), comparator and outcomes of the trial. These components of the research question are commonly referred to as the ‘PICO question’.^4^ Multiple neonatal research questions are potentially amenable to RCTs; however, trials must be selected carefully because they are expensive and often require large numbers of the target population to participate, which can have co-enrolment consequences for other research. There is a clear need to identify and prioritise research questions; this can be achieved through priority setting involving key stakeholders.

Priority setting partnerships have been used throughout perinatal medicine and demonstrate the value of involving key stakeholders such as parents, patients and healthcare professionals alongside researchers.^5^ Such partnerships, notably led by the James Lind Alliance,^6^ have addressed topics including preterm birth,^7 8^ stillbirth,^9^ childhood neurological conditions,^10^ diabetes in pregnancy^11^ and pregnancy hypertension^12^ to help guide future research directions. These priority setting partnerships have been invaluable for identifying broad research themes but are rarely detailed enough to yield specific research questions suitable for interventional trials.

To reduce research waste, clinical uncertainties should be evaluated wherever possible through definitive randomised trials with sufficient power and methodological robustness to provide answers that inform clinical practice.^13^ This initiative aimed to identify and prioritise neonatal research questions suitable for evaluation in definitive interventional trials using the more detailed and granular PICO format. Through a transparent, reproducible and inclusive methodology, this process aimed to support development and commissioning of practice-changing interventional trials in neonatology, to address those questions most important to healthcare professionals, parents and researchers.

## Methods

A steering group guided the development and conduct of this work, including representatives from academia, key neonatal organisations, clinical neonatology, neonatal nursing, allied healthcare professionals (AHPs), statisticians and parents with experience of neonatal care (online supplemental text 1). The protocol was designed collaboratively and published prior to data analysis.^14^

10.1136/fetalneonatal-2023-325504.supp1 Supplementary data

### Scope

The scope of the prioritisation process was developed and agreed by the steering group. Research questions had to be relevant to high-income neonatal care settings and proposed interventions expected to be delivered by neonatal teams. This included care provided on delivery suites, neonatal units, transitional care units and postnatal wards, during neonatal transport and within the community by neonatal teams after inpatient neonatal care. Research at pre-RCT stages of the translational pipeline was outside the scope of the process.

### Overview

Established research priority setting methodology as outlined by the James Lind Alliance was modified by the steering group to focus on detailed PICO questions, rather than general research themes or outcomes.

Phase 1: identification of neonatal research questions suitable for addressing in RCTs.

Phase 2: review of submitted neonatal research questions to remove duplicate questions and previously answered questions.

Phase 3: prioritisation of neonatal research questions by all relevant stakeholders using a three-round eDelphi process.

Phase 4: dissemination of ranked list of research questions in PICO format.

### Participants

The following participant groups were recruited for involvement in both the question submission and the Delphi prioritisation:

1. Clinicians involved in neonatal care: neonatologists, paediatricians, trainee doctors, neonatal nurses and advanced neonatal nurse practitioners were contacted through professional organisations including the British Association of Perinatal Medicine (BAPM), the Neonatal Nurses Association and the Neonatal Society, through organisational websites, direct email correspondence with members, regional teaching and meetings and social media.
2. AHPs involved in neonatal care: occupational therapists, physiotherapists, dietitians, speech and language therapists and clinical psychologists were contacted through the Association of Paediatric Chartered Physiotherapists, Royal College of Occupational Therapists, British Dietetic Association and Royal College of Speech and Language Therapists through websites, regional and national meetings and social media.
3. Researchers: academics and researchers working within neonatology were contacted through the Neonatal Society, other existing research networks, regional and national meetings and through clinical trial units with a neonatal interest.
4. Parents and former neonatal patients: parents, former patients and family members with experience of neonatal care were contacted through the national care coordinator groups, Maternity Voices Partnerships, relevant charity and advocacy websites and through social media.

We requested and recorded basic background descriptive data from participants. By ongoing monitoring of these data throughout the study, we aimed to ensure representation across the different stakeholder groups and of diverse social and ethnic groups—targeting under-represented groups accordingly. Recruitment was international, with participants requested to have personal experience of neonatal care or research in high-income settings.

### Question design and submission

A bespoke platform for question submission was devised using ‘OnlineSurvey’ (Jisc Services Limited, UK) software, with iterative development and face validity testing from all steering group members. The platform guided participants through the practicalities of structuring questions in the PICO format. We used categorical variables for gestational age and geographical location in the population (P) domain alongside a free-text field and used free-text fields for intervention (I) and comparison (C) domains. Outcomes could be selected from a categorical variable populated with the Core Outcomes in Neonatology^15^ or through a free-text field (online supplemental figure 1). We recognised generating research questions using the PICO structure could be challenging for some participants; therefore, the following strategies were developed:

10.1136/fetalneonatal-2023-325504.supp2 Supplementary data

1. An example PICO question based on a well-known neonatal trial was displayed on the question submission platform.
2. Pre-recorded video resources were developed for the BAPM website, showing members of the steering group putting together a PICO question relevant to their branch of practice. Links to these resources were included on the question submission software.
3. Two BAPM-supported webinars were held, explaining the development of PICO questions: one targeted towards all participants and one specifically designed to support parents and former patients led by a parent representative.

We contacted other groups who had undertaken neonatal priority setting work (for example, related to neonatal transport) directly and included relevant research questions in PICO format.

Each submitted question was reviewed by two independent members of the steering committee to remove questions that were incomplete, duplicate, out of scope, unclear or already answered, prior to progression to the eDelphi.

### Prioritisation process

All eligible research questions were entered into a three-round eDelphi survey using ‘DelphiManager’ (Comet Initiative Delphi Manager, University of Liverpool, UK) software, to establish a consensus as to their importance. Participants were asked to rank each research question on a 9-point Likert scale with 1 representing ‘no importance’ and 9 representing ‘critical importance’. After completion of round one, participants could suggest additional questions in PICO format which underwent the same review process as existing questions and were added to the second round of the eDelphi. Due to the large number of research questions, the second and third rounds of the eDelphi were limited to the top 75 and 50 ranked questions, respectively, to help minimise attrition rates. In the third round, the ranking by individual stakeholder group was displayed using the DelphiManager software (online supplemental figure 2) so that participants could choose to alter their answers based on the views of others. Analysis involved results being ranked by mean scores across all the stakeholder groups combined.

10.1136/fetalneonatal-2023-325504.supp3 Supplementary data

### Parental and former patient involvement

To maximise accessibility for non-clinical participants, guidance was provided by the study steering group parent representative throughout the prioritisation process. Advice was sought from key advocacy organisations such as Bliss to determine how best to meaningfully involve parents and ex-neonatal patients while keeping questions specific enough to be addressed in interventional trials. In addition to the well-attended focused parental webinar already described, videos of sample PICO questions were recorded by different stakeholders including a parent with experience of neonatal care. Publicity for involvement in the Delphi stages of the questionnaire was supported by a range of organisations including Maternity Voices Partnerships, local parent groups and relevant advocacy and charitable groups.

## Results

The national neonatal priority setting partnership was completed as outlined in the study protocol.^14^

### Question development

**Figure 1:**
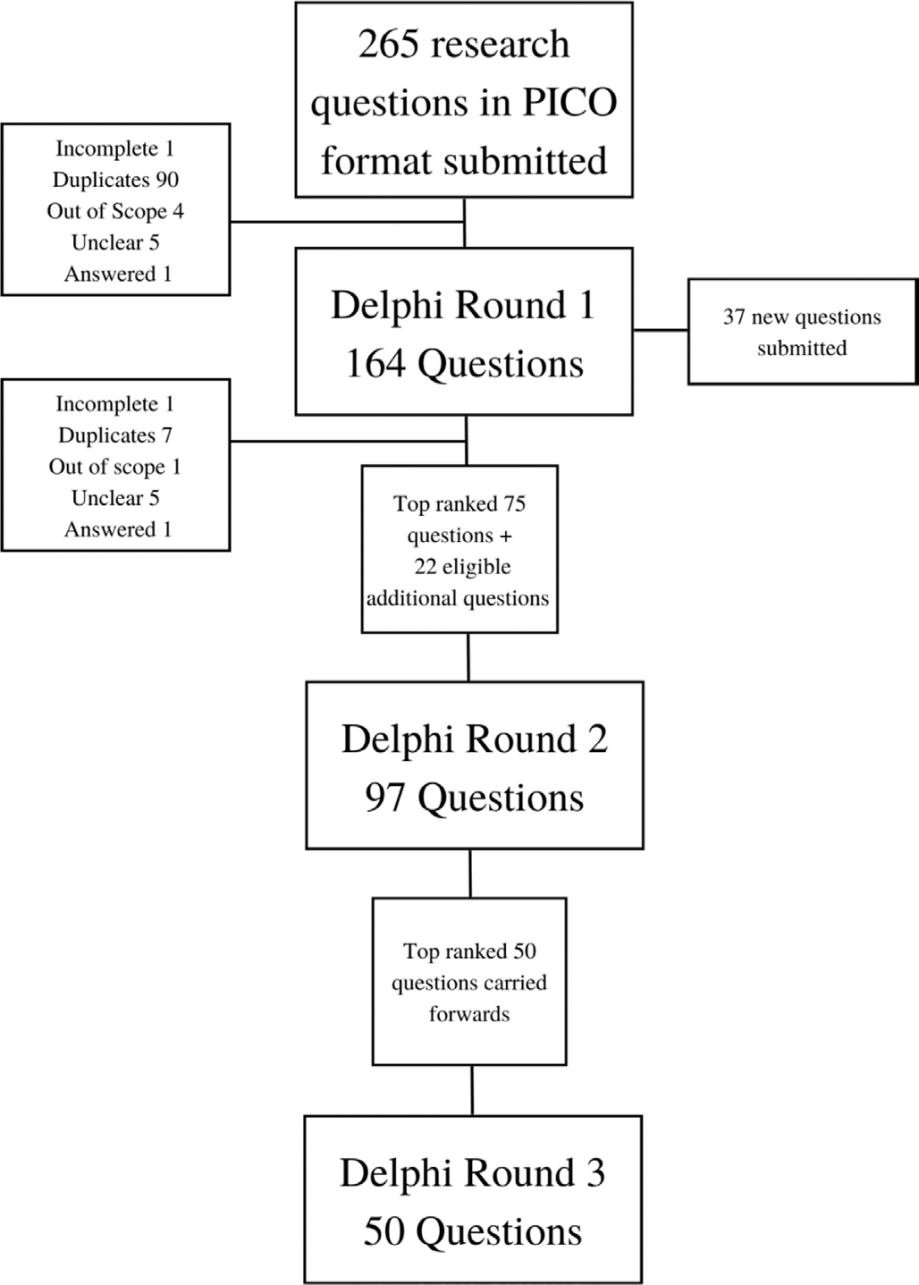
Flow chart of question identification and eDelphi consensus process. PICO, population, intervention, comparison, outcome.

Two hundred and sixty-five questions were submitted in PICO format during the 1-month submission period, from a total of 108 participants. The most common themes for questions were feeding and nutrition (20%) and family integrated care (20%). Stakeholder group breakdown was 11% parents, 4% nurses, 49% doctors, 11% AHPs, 15% researchers and 11% other (table 1). The flow of research questions throughout the study is represented in figure 1.

**Table 1:** Participant characteristics

	Question submission	eDelphi survey	
Total number of participants	265	144	
Stakeholder group			
Parents/former patients	30 (11%)	13 (9%)	
Nurses/allied healthcare professionals	38 (14%)	42 (29%)	
Doctors/researchers	169 (64%)	89 (62%)	
Other	28 (11%)	0 (0%)*	
Gender			
Male	73 (28%)	41 (29%)	
Female	163 (62%)	103 (71%)	
Prefer not to say	29 (10%)	0 (0%)*	
Ethnicity			Census^2021^
Asian/Asian British	24 (9%)	20 (14%)	9.30%
Black/African/Caribbean/black British	8 (3%)	4 (3%)	4.00%
Mixed/multiple ethnic	10 (4%)	5 (3%)	2.90%
White	181 (68%)	108 (75%)	81.70%
Other	42 (16%)	7 (5%)	2.10%

*‘Other’ not included as an option in the eDelphi survey.

### eDelphi survey

**Figure 2:**
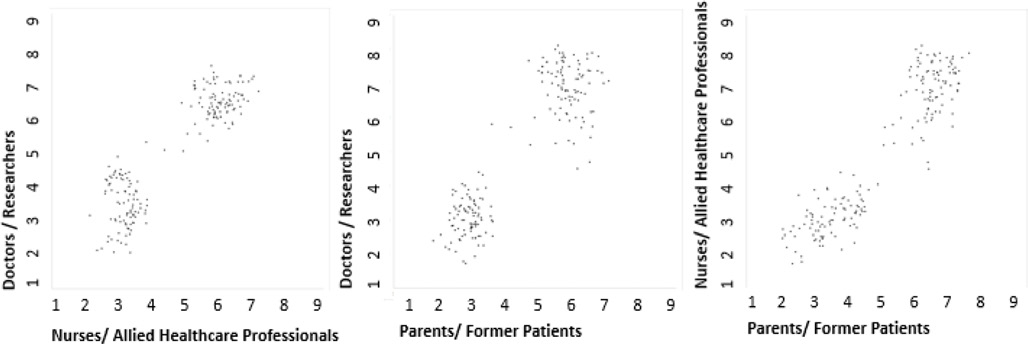
Prioritisation of research questions by stakeholder groups across round two of the eDelphi.

The three-phase online Delphi survey opened in May and was completed in August 2022; over 200 participants registered their interest. One hundred and sixty-four questions were eligible for entry into the first round of the survey which was completed by 144 participants. Raw scores displayed a bimodal distribution when compared across stakeholder groups with a clear consensus regarding those deemed more important (figure 2). Attrition rates across the three rounds were highest between rounds one and two (21.5%) and lower between rounds two and three (6.2%). Within individual stakeholder groups, attrition rates were highest in parents and former patients (53.9%), followed by nursing and AHPs (47.5%) and doctors and researchers (13.7%).

**Figure 3:**
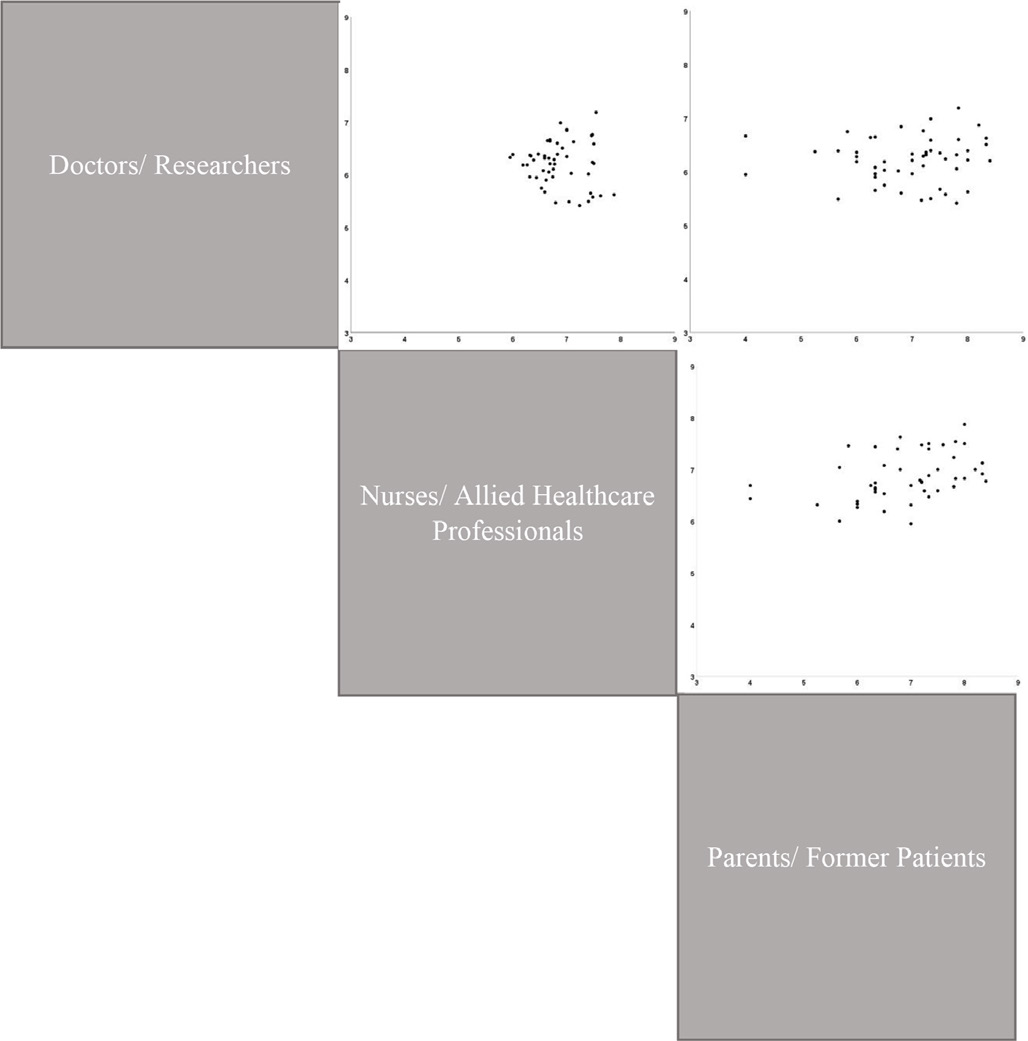
Stakeholder variability on round three of the eDelphi. Pairwise comparisons by stakeholder group of the ranked mean scores from round three for each outcome. Multiple pairwise comparisons presented together to aid visualisation. Comparisons arranged so that they are vertically or horizontally aligned to the stakeholder group label.

Thirty-seven new questions were submitted during round one of the eDelphi; 22 of these were deemed eligible for entry into round two. The results of round three displayed similar concordance between stakeholder groups, although with a higher consensus between the clinical groups (figure 3) than between clinical and parent/patient groups.

### Final list of prioritised research questions

All eligible questions were amalgamated into a final list of prioritised research questions and can be viewed in online supplemental text 2. The top 10 most highly ranked questions are displayed in table 2.

10.1136/fetalneonatal-2023-325504.supp4 Supplementary data

**Table 2:** Final list of top 10 prioritised research questions

Ranking	Question	Final mean score
1	Does routine fortification of human milk feeds improve necrotising enterocolitis and long-term neurodevelopmental outcomes in preterm babies?	7.305
2	In preterm and term babies requiring resuscitation, does intact cord resuscitation improve survival and brain injury compared with standard resuscitation with early cord clamping?	6.990
3	In babies diagnosed with necrotising enterocolitis, does earlier surgical intervention improve survival, brain injury and quality of life compared with standard practice?	6.959
4	Does therapeutic hypothermia (cooling) reduce brain injury and improve general cognition in babies with mild hypoxic ischaemic encephalopathy compared with standard care?	6.920
5	In extremely preterm infants (<28 weeks’ gestation at birth), should we routinely use non-invasive positive pressure ventilation or continuous positive airway pressure as the primary mode of respiratory support to improve survival and reduce bronchopulmonary dysplasia?	6.867
6	Is early breastmilk fortification or late breastmilk fortification superior with regard to outcomes such as necrotising enterocolitis in preterm babies?	6.857
7	In preterm babies, do probiotics improve survival, sepsis and necrotising enterocolitis?	6.838
8	Does human-derived milk fortifier rather than bovine-derived milk fortifier improve outcomes such as necrotising enterocolitis in preterm babies?	6.838
9	In very preterm infants at delivery, does physiological-based cord clamping (ie, stabilisation or resuscitation with the cord intact and only clamping when heart rate is >100 beats/min and oxygen saturation >85% in an inspired oxygen concentration of <0.4) versus time-based clamping at 60 s (or earlier if stabilisation or resuscitation is needed) increase survival without disability?	6.714
10	In preterm infants with insufficient maternal milk available, does the use of pasteurised human milk (donor) as compared with preterm formula reduce necrotising enterocolitis requiring surgery and improve 2-year neurodevelopmental outcomes?	6.705

## Discussion

Using a robust, reproducible consensus methodology, we have identified and prioritised 186 neonatal research questions suitable for definitive interventional clinical trials. Through involvement of a broad range of stakeholders, the results are generalisable to the wider neonatal community in the UK. These results should inform the design of practice-changing clinical trials to ensure such trials address clinically relevant research questions and avoid contributing to research waste.^16^

This neonatal research priority setting partnership builds on previous priority setting work by Duley *et al,* 7 which identified 15 broad themes of interest for research related to preterm birth, such as reducing infections, necrotising enterocolitis (NEC) and bronchopulmonary dysplasia. The detailed research questions prioritised in this work align closely with these broad research themes, particularly the importance of preventing NEC. Our work widens the scope by including research questions relating to all infants requiring neonatal care and is distinct in providing more granular and detailed research questions suitable for answering in practice-changing interventional trials.

A strength of this project is the large numbers of participants: over 200 people from several different high-income countries identified and ranked research questions. Additional strengths include ongoing parent representation with the use of specially designed training materials and question submission software supporting involvement in designing PICO questions. Finally, the use of a well-established, transparent eDelphi methodology ensures that this process was robust and reproducible for use in future initiatives. This approach could be used to identify and prioritise research questions suitable for other methodologies such as qualitative research.

A limitation of this work was attrition during the eDelphi survey, which was most notable among parents and former patients. Ensuring ongoing parent, patient or public participation in Delphi surveys is well recognised to be challenging.^17^ Attrition rates are lower if patient recruitment is through treatment centres rather than patient charities and advocacy organisations^18^; however, in previous neonatal priority setting work,^7^ neonatal unit-based recruitment of parents was also challenging,^8^ hence was not pursued during this study. We recognise that the lower levels of participation from parents and former patients may have influenced our final results, but a clear bimodal distribution of rankings with significant clustering of the same top-ranked and lower-ranked questions was consistent across all stakeholder groups. Given the small differences seen in mean rankings among highly prioritised research questions, these should be considered together as a group, with less emphasis on exact position in the ranking (online supplemental table 2) when planning future research.

We recognised at the outset that meaningful involvement in prioritisation required complex medical and technical knowledge of neonatal medicine, and that this knowledge may not be easily accessible to parents and ex-neonatal patients. We did however endeavour to include parents and ex-neonatal patients as they are key stakeholders in research designed to resolve uncertainties about the use of existing treatments. A different process would be needed to prioritise RCTs of emerging new therapies at earlier stages of translation. Following engagement with our parental representative and the organisation charity Bliss, we attempted specific and targeted parental prioritisation using plain English summaries of the most highly ranked questions. However, even this approach was considered inappropriate by our parent representative and charity partners who concluded that for parental involvement to be truly meaningful, it should be addressed by a more targeted qualitative approach focused on smaller numbers of research questions. Therefore, while robust health professional input was obtained from the full range of neonatal clinical and allied professions, this process should be considered less representative of parent and ex-neonatal patient views.

Priority setting work is becoming more widespread, with a recent scoping review showing that health-related topics encompassed 93% of all priority setting projects completed by the end of 2020.^19^ To our knowledge, the work to date has focused on identification of research themes or areas of interest, rather than targeting questions structured in a PICO format. Some studies have reformatted themes into PICO questions^20^; however, these have then been prioritised through a consensus group workshop, rather than with widespread stakeholder involvement. We believe our study is among the first to solely invite submission and prioritisation of research questions in PICO format suitable for answering in definitive interventional trials. Although outside the scope of this study, we recognise that well-designed RCTs should include qualitative elements to ensure that parental and patient experiences are captured, improving consent processes and overall success.

Future steps include sharing these prioritised research questions with clinical trial funders through existing commissioning processes. Our study methods and training materials strove to support detailed PICO question formation; however, we recognise some questions will require further refinement prior to evaluation in perinatal and neonatal adaptive trial platforms. Utilisation of priority setting results by research funders is expanding rapidly and there is variation in the methods used.^21^ Within high-income neonatal settings such as the National Health Service, this list will provide inspiration for the planning, design, funding and performance of future practice-changing trials.

## Conclusion

We have identified a prioritised list of detailed neonatal research questions suitable for addressing in interventional trials. Involvement of a broad range of stakeholder groups has ensured relevance to the wider neonatal community. The results of this prioritisation process will help guide future funding and development of interventional trials to ensure that they address questions of clinical import, change clinical practice and reduce research waste.

## Data Availability

All data relevant to the study are included in the article or uploaded as supplemental information. All data relevant to this study are uploaded as supplemental information.
