# Supplementary material for: National priority setting partnership using a Delphi consensus process to develop neonatal research questions suitable for practice-changing randomised trials in the UK
Source: Arch Dis Child Fetal Neonatal Ed. 2023 Apr 24;108(6):569–74. doi: 10.1136/archdischild-2023-325504 (PMC10646876; doi:10.1136/archdischild-2023-325504)
Supplement: Supplementary data [file fetalneonatal-2023-325504supp001.pdf]

Online supplementary eText 1: Members of the Neonatal Priority Setting Partnership Steering Group.

**Steering Group Member**	**Role and affiliation**
Cheryl Battersby (CB)	Academic Neonatologist, British Association of Perinatal Medicine (BAPM) Data/Informatics lead & member of NIHR prioritisation committee.
James Boardman (JPB)	Professor of Neonatal Medicine and immediate past president of the Neonatal Society.
Elaine Boyle (EB)	Professor of Neonatal Medicine and Chair of the National Institute for Health Research Neonatal Clinical Studies Group.
William Carroll (WC)	Consultant Paediatrician and Royal College of Paediatrics and Child Health (RCPCH) officer for Research.
Jon Dorling (JD)	Professor of Paediatrics, Neonatal Consultant and BAPM research lead.
Kate Dinwiddy (KD)	Chief Executive of BAPM.
Katie Evans (KE)	Project Co-ordinator and Honorary Clinical Research Fellow in Neonatal Medicine.
Chris Gale (CG)	Professor of Neonatal Medicine and Neonatal Society Meeting Secretary.
Katie Gallagher (KG)	Academic Neonatal Nurse and Neonatal Nurses Association representative.
Pollyanna Hardy (PH)	Clinical Trials Statistician and Director of National Perinatal Epidemiology Unit Clinical Trials Unit.
Emma Johnston (EJ)	Parent representative and Parents and Family engagement Lead with the Thames Valley and Wessex Operational Delivery Network.
Helen Mactier (HM)	Consultant Neonatologist, Honorary Clinical Associate Professor and immediate past president of BAPM.
Claire Marcroft (CM)	Neonatal Physiotherapist and Allied Health Professionals Representative.
James Webbe (JW)	Trainee representative and Neonatal Medicine Trainee.
