# Supplementary material for: National priority setting partnership using a Delphi consensus process to develop neonatal research questions suitable for practice-changing randomised trials in the UK
Source: Arch Dis Child Fetal Neonatal Ed. 2023 Apr 24;108(6):569–74. doi: 10.1136/archdischild-2023-325504 (PMC10646876; doi:10.1136/archdischild-2023-325504)
Supplement: Supplementary data [file fetalneonatal-2023-325504supp002.pdf]

Online Supplementary eFigure 1: Question submission software designed to support participants with submission of research questions in a population, intervention, comparison, outcome (PICO) structure.

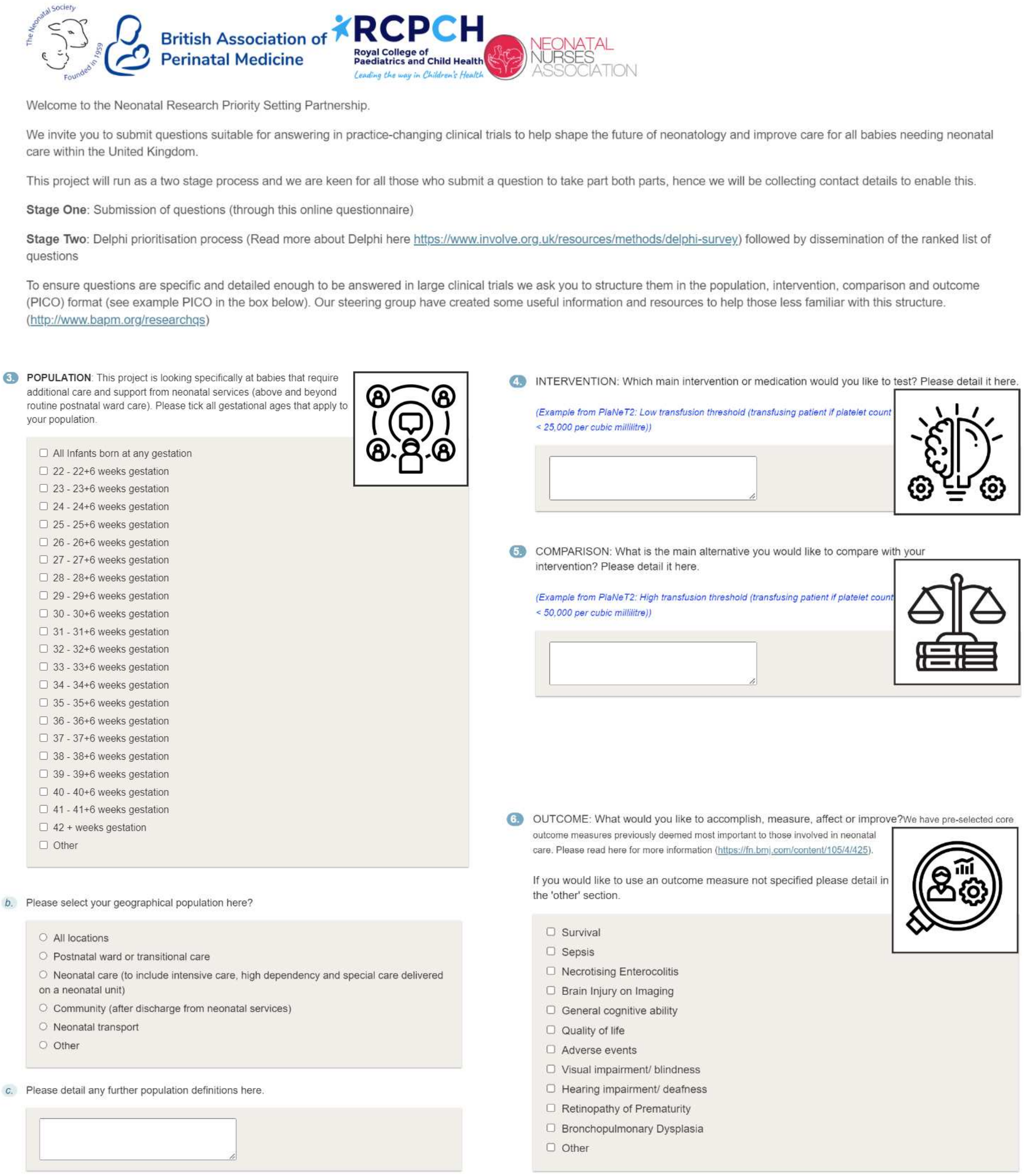

Welcome to the Neonatal Research Priority Setting Partnership.

We invite you to submit questions suitable for answering in practice-changing clinical trials to help shape the future of neonatology and improve care for all babies needing neonatal care within the United Kingdom.

This project will run as a two stage process and we are keen for all those who submit a question to take part both parts, hence we will be collecting contact details to enable this.

Stage One: Submission of questions (through this online questionnaire)

Stage Two: Delphi prioritisation process (Read more about Delphi here https://www.involve.org.uk/resources/methods/delphi-survey) followed by dissemination of the ranked list of questions

To ensure questions are specific and detailed enough to be answered in large clinical trials we ask you to structure them in the population, intervention, comparison and outcome (PICO) format (see example PICO in the box below). Our steering group have created some useful information and resources to help those less familiar with this structure. (http://www.bapm.org/researchqs)

3. **POPULATION**: This project is looking specifically at babies that require additional care and support from neonatal services (above and beyond routine postnatal ward care). Please tick all gestational ages that apply to your population.

- ☐ All Infants born at any gestation
- ☐ 22 - 22+6 weeks gestation
- ☐ 23 - 23+6 weeks gestation
- ☐ 24 - 24+6 weeks gestation
- ☐ 25 - 25+6 weeks gestation
- ☐ 26 - 26+6 weeks gestation
- ☐ 27 - 27+6 weeks gestation
- ☐ 28 - 28+6 weeks gestation
- ☐ 29 - 29+6 weeks gestation
- ☐ 30 - 30+6 weeks gestation
- ☐ 31 - 31+6 weeks gestation
- ☐ 32 - 32+6 weeks gestation
- ☐ 33 - 33+6 weeks gestation
- ☐ 34 - 34+6 weeks gestation
- ☐ 35 - 35+6 weeks gestation
- ☐ 36 - 36+6 weeks gestation
- ☐ 37 - 37+6 weeks gestation
- ☐ 38 - 38+6 weeks gestation
- ☐ 39 - 39+6 weeks gestation
- ☐ 40 - 40+6 weeks gestation
- ☐ 41 - 41+6 weeks gestation
- ☐ 42 + weeks gestation
- ☐ Other

b. Please select your geographical population here?

- ○ All locations
- ○ Postnatal ward or transitional care
- ○ Neonatal care (to include intensive care, high dependency and special care delivered on a neonatal unit)
- ○ Community (after discharge from neonatal services)
- ○ Neonatal transport
- ○ Other

c. Please detail any further population definitions here.

4. INTERVENTION: Which main intervention or medication would you like to test? Please detail it here.

(Example from PlaNeT2: Low transfusion threshold (transfusing patient if platelet count < 25,000 per cubic millilitre))

5. COMPARISON: What is the main alternative you would like to compare with your intervention? Please detail it here.

(Example from PlaNeT2: High transfusion threshold (transfusing patient if platelet count < 50,000 per cubic millilitre))

6. OUTCOME: What would you like to accomplish, measure, affect or improve? We have pre-selected core outcome measures previously deemed most important to those involved in neonatal care. Please read here for more information (https://fn.bmj.com/content/105/4/425).

If you would like to use an outcome measure not specified please detail in the 'other' section.

- ☐ Survival
- ☐ Sepsis
- ☐ Necrotising Enterocolitis
- ☐ Brain Injury on Imaging
- ☐ General cognitive ability
- ☐ Quality of life
- ☐ Adverse events
- ☐ Visual impairment/ blindness
- ☐ Hearing impairment/ deafness
- ☐ Retinopathy of Prematurity
- ☐ Bronchopulmonary Dysplasia
- ☐ Other
