# Supplementary material for: National priority setting partnership using a Delphi consensus process to develop neonatal research questions suitable for practice-changing randomised trials in the UK
Source: Arch Dis Child Fetal Neonatal Ed. 2023 Apr 24;108(6):569–74. doi: 10.1136/archdischild-2023-325504 (PMC10646876; doi:10.1136/archdischild-2023-325504)
Supplement: Supplementary data [file fetalneonatal-2023-325504supp003.pdf]

Online Supplementary eFigure 2: Example of Round 3 eDelphi view.

Different Stakeholder Groups average rankings were colour coded (Purple for parents/ former patients, Orange for nurses/ allied healthcare and grey for doctors/ researchers) . This was explained to participants in the key above each speciality domain and also in the introductory paragraph of the priority setting exercise.

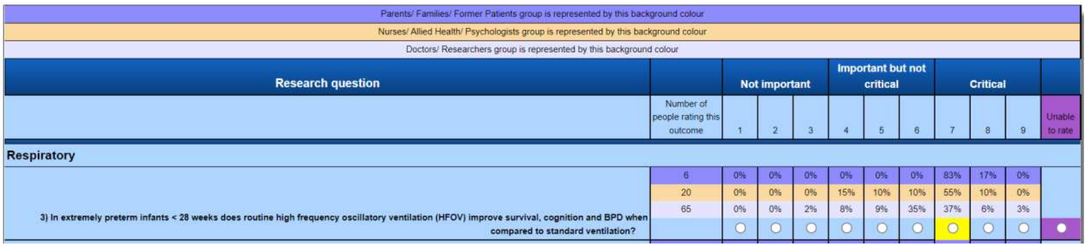

Parents/ Families/ Former Patients group is represented by this background colour											
Nurses/ Allied Health/ Psychologists group is represented by this background colour											
Doctors/ Researchers group is represented by this background colour											
Research question		Not important			Important but not critical			Critical			
	Number of people rating this outcome	1	2	3	4	5	6	7	8	9	Unable to rate
Respiratory											
3) In extremely preterm infants < 28 weeks does routine high frequency oscillatory ventilation (HFOV) improve survival, cognition and BPD when compared to standard ventilation?	6	0%	0%	0%	0%	0%	0%	83%	17%	0%	
	20	0%	0%	0%	15%	10%	10%	55%	10%	0%	
	65	0%	0%	2%	8%	9%	35%	37%	6%	3%	

Bronchopulmonary Dysplasia (BPD)
