# Supplementary material for: National priority setting partnership using a Delphi consensus process to develop neonatal research questions suitable for practice-changing randomised trials in the UK
Source: Arch Dis Child Fetal Neonatal Ed. 2023 Apr 24;108(6):569–74. doi: 10.1136/archdischild-2023-325504 (PMC10646876; doi:10.1136/archdischild-2023-325504)
Supplement: Supplementary data [file fetalneonatal-2023-325504supp004.pdf]

Online Supplementary eText 2: Full list of prioritised research questions.

Final mean scores displayed by individual stakeholder groups alongside final mean scores.

<u>Highly Prioritised Questions</u>

Rank	Question	Doctors/ Researchers	Nurses/ AHPs	Parents/ Former Patients	Mean Score
	**Round 3**				
1	Does routine fortification of human milk feeds improve necrotising enterocolitis and long-term neurodevelopmental outcomes in preterm babies?	7.187	7.542	7.833	**7.305**
2	In preterm and term babies requiring resuscitation; does intact cord resuscitation improve survival and brain injury compared to standard resuscitation with early cord clamping?	6.987	6.882	7.333	**6.990**
3	In babies diagnosed with necrotising enterocolitis does earlier surgical intervention improve survival; brain injury and quality of life compared to standard practice?	6.867	7.000	8.200	**6.959**
4	Does therapeutic hypothermia (cooling) improve brain injury and general cognition in babies with mild hypoxic ischaemic encephalopathy (HIE) compared to standard care?	6.763	7.474	7.200	**6.920**
5	In extremely preterm infants < 28 weeks should we routinely use non-invasive positive pressure ventilation (NIPPV) or continuous positive airway pressure (CPAP) as the primary mode of respiratory support to improve survival and reduce bronchopulmonary dysplasia (BPD)?	6.842	7.000	6.800	**6.867**
6	Is early breastmilk fortification or late breastmilk fortification superior with regards to outcomes such as necrotising enterocolitis in preterm babies?	6.767	7.458	5.822	**6.857**
7	In preterm babies do probiotics improve survival; sepsis and necrotising enterocolitis?	6.627	7.125	8.333	**6.838**
8	Does human-derived milk fortifier rather than bovine-derived milk fortifier improve outcomes such as necrotising enterocolitis in preterm babies?	6.587	7.500	7.333	**6.838**
9	In very preterm infants at delivery does physiological based cord clamping (i.e. stabilisation or resuscitation with the cord intact and only clamping when heart	6.600	6.824	7.833	**6.714**

	rate is> 100 beats per minute and oxygen saturation >85% in FiO2 <0.4) versus time-based clamping at 60 seconds (or earlier if stabilisation or resuscitation is needed) increase survival without disability?				
10	In preterm infants with insufficient maternal milk available; does the use of pasturised human milk (donor) as compared with preterm formula reduce necrotising enterocolitis requiring surgery and improve two-year neurodevelopmental outcomes?	6.507	6.917	8.333	**6.705**
11	In any baby with seizures does levetiracetam improve need for second-line anti-convulsants when compared to phenobarbitone?	6.649	6.643	6.333	**6.637**
12	In term infants with a bilious vomit who are assessed by a senior neonatologist as being well; does transfer to a specialised unit for urgent upper gastrointestinal contrast improve survival; quality of life and adverse events compared to close clinical monitoring by the local neonatal team?	6.640	6.688	6.250	**6.632**
13	In babies above 34 weeks gestation on the postnatal ward; is a blood glucose threshold of 2.0mmol/L non-inferior to a blood glucose threshold of 2.6mmol/L with regards to adverse events?	6.667	6.688	4.000	**6.585**
14	In infants born extremely preterm (< 28 weeks) does 1 to 1 nursing care until 28 weeks corrected gestation improve survival and all core neonatal outcomes compared to standard nursing allocations based on intensive care support required?	6.392	6.826	8.000	**6.582**
15	In babies born preterm does a post-discharge home intervention package of brain stimulation exercises improve neurodevelopment when compared to standard care?	6.216	7.500	8.000	**6.551**
16	Does therapeutic hypothermia (cooling) improve brain injury and long term neurodevelopmental outcomes in preterm infants (> 30 weeks) who have suffered a hypoxic injury?	6.237	7.474	7.600	**6.540**
17	In infants with a prenatally diagnosed gastrointestinal anomaly does planned delivery in a unit with co-located neonatal surgical unit (no ambulance transfer required) improve survival; parental experience and adverse events?	6.347	7.000	7.500	**6.505**
18	In preterm babies does high dose caffeine (10-20mg/kg/day) improve survival; brain injury and cognition compared to low dose caffeine (5mg/kg/day)?	6.316	6.667	7.800	**6.455**

19	In preterm infants does enhanced monitoring from birth to 72 hours of life (with near infrared spectroscopy (NIRS); transcutaneous CO2; spO2; heart rate and arterial BP) improve core neonatal outcomes compared to standard monitoring?	6.395	6.471	7.333	**6.434**
20	In extremely preterm infants that are ventilator dependent does early dexamethasone treatment (2 weeks of age) compared to late dexamethasone treatment (4 weeks of age) improve survival; bronchopulmonary dysplasia and cognition?	6.360	6.588	7.250	**6.438**
21	In extremely preterm infants (< 28 weeks) do elective caesarean sections or vaginal deliveries result in better survival; brain injury and cognition?	6.203	6.773	8.400	**6.436**
22	If a mother is in preterm labour and expected to deliver imminently does administering a second dose of antenatal steroid early improve survival and bronchopulmonary dysplasia compared to standard care (not administering a second dose until the standard time period)?	6.289	6.765	7.200	**6.418**
23	In extremely preterm infants < 28 weeks does routine high frequency oscillatory ventilation (HFOV) improve survival; cognition and bronchopulmonary dysplasia when compared to standard ventilation?	6.316	6.588	7.250	**6.402**
24	In extremely preterm babies does restrictive total fluid (60; 90; 120; 150ml/kg/day) lead to improved survival and bronchopulmonary dysplasia when compared to liberal total fluid (90; 120; 150; 180; 200ml/kg/day)?	6.360	6.333	6.000	**6.344**
25	In extremely preterm infants does maintaining a midline head position for the first 72 hours of life improve survival and brain injury when compared to standard care?	6.014	7.400	6.750	**6.327**
26	In preterm babies who develop hyperglycaemia (>12mmol/L) should we treat with an insulin infusion or reduce the glucose infusion rate; to maximise growth and long term metabolic outcomes?	6.373	6.316	5.250	**6.316**
27	In preterm infants with echocardiographically confirmed persistent pulmonary hypertension of the newborn (PPHN) does inhaled nitric oxide improve survival; bronchopulmonary dysplasia and brain injury compared to no treatment?	6.211	6.688	7.000	**6.316**
28	In extremely preterm infants does on-demand haemodynamic assessment guided choice of inotrope therapy improve survival and other core outcomes compared to standard unit protocols?	6.387	6.000	5.667	**6.311**

29	In babies diagnosed with gastro-oesophageal reflux does the use of anti-reflux medications improve outcomes such as bronchopulmonary dysplasia; sepsis and quality of life when compared with non-pharmacological support?	6.107	6.750	7.200	**6.308**
30	In preterm babies showing feeding cues whilst on non-invasive respiratory support; does commencing oral feeding (compared to waiting) improve outcomes such as breastfeeding rates; oral aversion and reflux?	6.027	7.080	6.500	**6.302**
31	Does point of care ultrasound guided umbilical venous catheter (UVC) position adjustments reduce workload; X-ray exposure and adverse events compared to standard X-ray guided UVC position adjustments?	6.280	6.385	6.000	**6.289**
32	In preterm babies do high nutrient enteral/ parenteral nutrition strategies (macronutrients at upper end of recommended intakes) improve core neonatal outcomes when compared with lower nutrient enteral/ parenteral nutrition strategies (macronutrients at lower levels of recommended intakes) without adverse events?	6.329	5.950	7.000	**6.280**
33	Does increased staffing with dedicated allied health professionals or care support workers to support parents with caring for their baby improve quality of life; length of stay and parental wellbeing over standard staffing?	5.622	7.875	8.000	**6.279**
34	In preterm babies requiring parenteral nutrition; does early parenteral nutrition within 8 hours of birth improve survival and neurodevelopmental outcomes when compared to late parenteral nutrition after 48 hours of life?	6.053	6.667	7.800	**6.265**
35	In preterm infants does prophylactic hydrocortisone treatment from day 1 to 10 improve survival and other core neonatal outcomes compared to a placebo?	6.184	6.188	6.500	**6.198**
36	In ex-preterm babies with inguinal hernias; does repair prior to discharge improve adverse events such as incarceration; when compared with repair after discharge?	6.187	6.267	6.000	**6.191**
37	In extremely preterm infants should structured blood pressure management be aiming for a target of greater than 30mmHg or greater than gestational age; to improve survival and brain injury?	6.080	6.563	6.333	**6.170**
38	In preterm infants does starting treatment with early probiotics (<24 hours of birth) versus later probiotics (when tolerating trophic feeds) reduce risk of necrotising enterocolitis; sepsis; dysbiosis?	5.960	6.739	6.333	**6.154**

39	Does being born on a facilitative unit that promotes family centered care improve neurodevelopmental outcomes; length of stay and readmissions compared with traditional neonatal care?	5.575	7.480	7.600	**6.136**
40	In preterm babies does an embedded feeding team (comprising SALT; feeding advisor and dietetics) improve growth; breast-feeding rates; readmissions and parent-infant bonding as compared with standard care?	5.600	7.625	6.800	**6.125**
41	In moderately preterm infants (32-27 weeks) does immediate kangaroo mother care/ skin-to-skin for a minimum period of time per day improve breast-feeding rates; hypoglycaemia and autism spectrum disorders compared with standard care?	5.649	7.440	6.333	**6.114**
42	In all infants with cardiovascular instability; does access to targeted functional echocardiography improve outcomes such as survival; brain injury and kidney injury compared to standard care?	5.960	6.313	7.000	**6.053**
43	In all babies requiring admission to NICU at birth; does a delivery room cuddle with the parents improve survival; parental mental health and breast milk production when compared with no delivery room cuddle?	5.493	7.400	7.333	**6.047**
44	In babies with oesophageal atresia does routine placement of a transanastomotic tube (TAT) improve strictures; need for dilatations and need for rescue feeding method; when compared with no TAT placement?	5.899	6.615	6.333	**6.023**
45	In term babies over 48 hours of life; is a blood glucose threshold of 2.6mmol/L non-inferior to a blood glucose threshold of 3.0mmol/L for adverse events; cognition and breastfeeding rates?	5.947	6.438	4.000	**5.968**
46	In term babies receiving therapeutic hypothermia (cooling) does early parenteral nutrition improve survival and cognition when compared to only commencing parenteral nutrition after one week if feeds cannot be established?	5.671	6.591	7.500	**5.941**
47	In infants < 32 weeks gestation with patent ductus arteriosus (PDA) does artificial closure (ligation or medical) improve survival and bronchopulmonary dysplasia compared with supportive management only?	5.747	6.529	6.500	**5.917**
48	In all babies admitted to NICU; does a routine clinical psychologist assessment and ongoing support with weekly parent groups improve long term neonatal outcomes and parental mental health; compared to no psychology interventions?	5.486	7.040	5.667	**5.867**

49	In infants requiring higher level care does being delivered at a level three NICU improve survival; bronchopulmonary dysplasia and adverse events in comparison to postnatal transfer from a different unit?	5.467	6.792	7.167	**5.867**
50	In preterm infants; does adopting a 'golden hour' approach completing all procedures within one hour of birth improve survival; BPD and cognition when compared to standard care?	5.413	7.235	7.800	**5.856**
	**Round 2**				
51	Does the support of a dedicated specialist lactation consultant on NICU from admission to discharge improve growth; breast-milk feeding and maternal mental health?	5.751	6.676	6.889	**6.080**
52	In extremely preterm infants who received a dose of surfactant at birth do repeated doses of surfactant at 48 and 72 hours improve survival and bronchopulmonary dysplasia when compared to standard care?	5.812	6.500	7.813	**6.078**
53	For all infants requiring neonatal care does a formal multi-disciplinary discharge pathway with nationally approval parental information and tailored education sessions reduce readmission; A&E attendances and improve parental wellbeing compared to standard local discharge pathways?	5.467	7.100	7.294	**6.075**
54	Does supplementation with breast milk fortifier after discharge or term age improve growth; breast-feeding rates and long term outcomes in preterm babies?	5.909	6.418	6.111	**6.054**
55	Does the use of loperamide post stoma formation improve parenteral nutrition related liver disease; line sepsis and length of stay when compared to placebo?	5.968	6.000	7.500	**6.049**
56	In babies diagnosed with necrotising enterocolitis does remote ischaemic condition improve survival; necrotising enterocolitis and duration of parenteral nutrition compared to standard care?	5.808	6.600	7.091	**6.043**
57	Does a psychology intervention supporting staff with regular reflective practice and psychoeducation improve staff sickness; staff retention and staff mental health issues compared to no psychological intervention for staff?	5.570	7.014	6.118	**6.042**
58	In preterm infants receiving less invasive surfactant administration does use of pharmacological methods alongside environmental measures improve success rates and comfort when compared to environmental measures alone (swaddling/ sucrose)?	5.868	6.288	6.556	**6.004**

59	In preterm babies receiving donor milk due to insufficient mothers milk; is it superior to switch to preterm formula once on full feeds or wait until term corrected gestational age?	5.885	6.254	6.125	**5.996**
60	In preterm babies with chronic lung disease does extending caffeine therapy until term (rather than standard care of discontinuing around 34 weeks corrected gestation) improve survival and cognition?	5.783	6.077	7.889	**5.992**
61	In preterm babies requiring parenteral nutrition does higher range lipid (fats) intake or lower range lipid (fats) intake improve growth and long term metabolic outcomes?	6.097	5.827	5.231	**5.987**
62	In preterm infants does high dose vitamin D supplementation (>800 units) improve metabolic bone disease when compared to low dose vitamin D supplementation (< 400 units)?	5.981	6.000	5.909	**5.982**
63	In preterm babies whose mothers would like to establish exclusive breast-feeding; does exposure to routine bottle feeding reduce breast-feeding success compared to exposure to routine nasogastric feeding?	5.661	6.565	6.611	**5.969**
64	Does dopamine vs adrenaline use for hypotension in preterm infants result in improved survival; neurodevelopemental outcome at 24 months corrected age and reduced morbidity (such as necrotising enterocolitis and bronchopulmonary dysplasia)	5.847	6.000	7.750	**5.957**
65	Does follow up of moderate to late preterm infants to 5 years improve school age outcomes and health?	5.486	6.800	7.000	**5.953**
66	In neonates with meconium ileus and obstruction or post-laparotomy with obstruction; does saline or N-acetylcysteine treatment improve speed of establishing feeds or reduce the need for subsequent laparotomy when compared to supportive care?	5.952	6.154	5.333	**5.940**
67	In preterm infants with established chronic lung disease on invasive/non-invasive respiratory support at term corrected does a weaning course of prednisolone and azithromycin prophlyaxis improve home oxygen use and duration; readmissions with respiratory illnesses and length of stay?	5.872	5.956	6.818	**5.932**
68	Does the use of a respiratory function monitor to guide tidal volumes at birth reduce short-term need for ventilation and long-term outcomes of bronchopulmonary dysplasia; brain injury and 2 year outcomes?	5.764	6.000	7.667	**5.929**

69	In extremely preterm infants < 28 weeks is assist control ventilation (with volume targeting) superior to SIMV (with volume targeting) with regards to survival; ventilation days and bronchopulmonary dysplasia?	5.795	6.105	7.000	**5.928**
70	In babies diagnosed with NEC does treatment with pentoxifylline improve survival; duration of PN and brain injury compared to standard care?	5.795	6.105	7.091	**5.928**
71	In preterm babies does targeting higher oxygen saturations of 92-97% lead to improved survival without complications such as NEC (when compared to standard care?	5.647	6.113	8.111	**5.918**
72	Does providing ongoing psychology support in the community as part of the routine discharge package improve quality of life; bonding and parental mental health compared to routine discharge with no psychology follow-up?	5.204	7.042	7.316	**5.914**
73	In term babies admitted to NICU for respiratory support would lowering the target saturations range to > 90% (rather than standard target) improve length of stay and duration of respiratory support without increasing adverse outcomes?	5.769	5.980	7.235	**5.911**
74	In extremely preterm infants with patent ductus arteriosus on a screening echocardiogram does early medical treatment (< 7 days) improve core neonatal outcomes compared with no treatment?	5.755	6.205	6.538	**5.891**
75	In preterm babies < 32 weeks with an oxygen requirement of over 40% does routine intubation and ventilation improve survival and bronchopulmonary dysplasia when compared to intubation based solely on clinical indications such as apnoea?	5.639	6.255	7.353	**5.877**
76	Does providing a bespoke psychological intervention to parents who witnessed a serious incident (either for their own or a different baby) improve parental trauma scores; parental mental health and quality of life compared to not providing bespoke support?	5.187	6.931	7.316	**5.876**
77	Does education for health professionals about the support needs of ethnically diverse families reduce increased neonatal mortality risk; when compared to standard staff training?	5.229	6.839	7.750	**5.872**
78	Does a weekly 'allied health professional' ward round with parents present improve parental satisfaction; when compared to allied health professional ward rounds without parental presence?	5.200	6.904	7.500	**5.861**

79	In babies with seizures does maintenance topiramate improve seizure control and brain injury when compared to phenobarbitone?	5.716	6.306	6.273	**5.851**
80	In extremely preterm infants with respiratory distress requiring surfactant administration is elective intubation and ventilation preferable to less-invasive-surfactant-administration to improve pneumothorax rates and bronchopulmonary dysplasia?	5.696	6.395	5.933	**5.837**
81	In babies on the NICU; do parent-led ward rounds improve quality of life; parental mental health and breast-feeding rates in comparison to standard clinician led ward rounds?	5.207	6.819	7.053	**5.834**
82	In babies with respiratory distress syndrome should a second dose of surfactant be given at 30% or 40% to improve bronchopulmonary dysplasia rates and other core neonatal outcomes?	5.740	5.750	7.400	**5.827**
83	Do preterm infants have lower scores on standardised Speech and Language scales at 18 months; 3 years and 5 years compared to term born infants demonstrating the need for fully funded; robust speech and language services at neonatal discharge?	5.331	6.403	7.625	**5.808**
84	In extremely preterm infants that are ventilator dependent on day 8 of life does a treatment course of nebulised budesonide as compared to placebo improve survival; bronchopulmonary dysplasia; and length of stay?	5.614	6.071	7.600	**5.798**
85	In babies born pre-term; who have bronchopulmonary dysplasia; does a standardised ventilation weaning plan improve duration of ventilation; length of hospital stay; growth; quality of life and neurodevelopmental outcomes at 12 and 24 months?	5.597	6.150	7.200	**5.794**
86	In babies with suspected hypoxic ischaemic encephalopathy in level 2 neonatal units; does the use of telemedicine to aid diagnosis improve long-term neurodevelopmental outcomes?	5.366	6.565	7.333	**5.760**
87	In very preterm infants at delivery does setting initial FiO2 at 0.6 versus 0.3 and targeting oxygen saturations of 80-85% at 5 mins and 85-95% at 10 mins or later increase survival without disability?	5.577	5.941	8.000	**5.725**
88	For parents of preterm infants does delivering detailed information of future health risks at discharge; improve quality of life; parental satisfaction and	5.099	6.300	7.375	**5.596**

	parental mental health; when compared to providing it at a later timepoint (for example 12 months corrected age)?				
89	In babies diagnosed antenatally with congenital diaphragmatic hernia; does the use of intranasal sedation for intubation at delivery; compared to no sedation lead to fewer intubation attempts; better cardiorespiratory stability and improved long term neurodevelopmental outcomes?	5.423	6.286	6.250	**5.596**
90	In preterm infants born at <28w gestation does regular screening for Ureaplasma colonisation and treatment where indicated; improve outcomes (bronchopulmonary dysplasia; length of stay)?	5.580	5.300	5.250	**5.530**
91	Does a structured package of malnutrition investigations improve outcomes such as nutritional deficiences; growth and core long-term outcomes?	5.211	5.792	7.500	**5.525**
92	In all babies requiring intubation; does the nasal route offer an acceptable alternative that results in fewer unplanned extubations?	5.356	5.500	6.667	**5.465**
93	In all infants (including preterm infants) with suspected hypoxic ischaemic encephalopathy; does intervention with therapeutic hypothermia (cooling) affect visual development; when compared to standard care?	4.814	6.409	7.667	**5.347**
94	Does a smoking cessation support package delivered in NICU by NICU staff reduce parental smoking relapse rates by 12 months post discharge?	4.986	5.500	6.000	**5.192**

Lower Prioritised Questions

**Rank**	**Question**	**Doctors/ Researchers**	**Nurses/ AHPs**	**Parents/ Former Patients**	**Mean Score**
95	Does regular ultrasound monitoring of umbilical lines to identify associated thrombus improve core neonatal outcomes?	4.861	5.000	5.200	**4.905**
96	Do breastfeeding preterm infants who receive routine daily vitamin K supplementation post-discharge have lower prevalence of vitamin K deficiency in infancy?	4.319	5.038	5.714	**4.590**

97	For all babies requiring neonatal care; does a formal nursing training package on length measurement improve reliability and validity of measurements when compared to standard care?	3.771	5.267	5.800	**4.295**
	**Round 1**				
98	Does referral to early intervention occupational therapy at the point of neonatal discharge improve cognition; quality of life and social-emotional outcomes at school age; compared to no routine occupational therapy referral?	5.539	6.500	7.222	**5.968**
99	In babies undergoing therapeutic hypothermia does midazolam sedation rather than standard care with morphine sedation improve brain injury and cognition?	5.723	6.556	6.500	**5.968**
100	Does dedicated physiotherapy support after discharge improve parental and infant mental health; compared to no physiotherapy support?	5.544	6.556	6.500	**5.966**
101	In extremely preterm infants that are ventilator dependent on day 8 of life and likely to need pressure support ventilation at 36 weeks corrected gestation does a treatment course of hydrocortisone; as compared to a treatment course of dexamethasone improve survival and bronchopulmonary dysplasia?	5.832	6.083	6.778	**5.940**
102	In babies diagnosed with necrotising enterocolitis does therapeutic hypothermia improve survival and brain injury when compared to standard care?	5.667	6.733	7.000	**5.939**
103	In extremely preterm infants does limiting routine physical examination to times of clinical/parental or nursing concerns improve survival and complications of prematurity when compared to daily routine examination on ward rounds?	5.583	6.767	6.571	**5.934**
104	In preterm infants with respiratory distress syndrome requiring surfactant therapy is the best mode of delivery an iGel or a less-invasive-surfactant-administration catheter to improve success rates and mechanical ventilation rates?	5.879	6.083	5.900	**5.917**
105	In preterm babies receiving nasogastric feeds; does routinely aspirating compared to not routinely aspirating improve time to full enteral feeds; growth and length of stay without adverse events?	6.104	5.417	5.600	**5.894**

106	When accessing lines does a sterile aseptic technique improve survival and sepsis over an aseptic non-touch technique?	5.819	5.818	6.750	**5.885**
107	Does a staff and parental training package on psychologically informed environments; trauma-informed care and compassion focused approaches improve core neonatal outcomes and parental mental health?	5.077	7.077	7.364	**5.883**
108	In infants with chronic lung disease does diuretic therapy improve home oxygen rates and neurodevelopmental outcomes when compared to no diuretics?	5.653	6.077	7.500	**5.869**
109	In preterm babies requiring parenteral nutrition does higher range energy (calorie) intake or lower range energy (calorie) intake improve growth and long term metabolic outcomes?	5.854	5.867	6.000	**5.865**
110	In all babies admitted to NICU; does a dedicated education training package covering unit familiarisation & protocols; parenting issues; financial support and bonding improve parental mental health alongside long-term neonatal outcomes; when compared to standard care?	5.434	6.410	6.818	**5.857**
111	In preterm babies does targeting tight control of weight gain along birth centile using calorific interventions improve outcomes when compared to allowing growth trajectory below birth centile (as per standard growth using population data)?	6.031	5.500	5.375	**5.852**
112	In all infants characterised as having circulatory failure; does the use of certain biomarkers improve survival and brain injury when compared to standard monitoring using blood pressure?	5.663	6.381	6.429	**5.847**
113	Does access to specialist neonatal respiratory physiotherapy (percussion/ treatment techniques) compared to neonatal staff training on physiotherapy techniques improve adverse events and neonatal outcomes?	5.440	6.381	6.429	**5.824**
114	In invasively ventilated extremely preterm infants is extubation to synchronised non-invasive positive pressure ventilation (NIPPV) or non-synchronised NIPPV superior when considering extubation success; survival and bronchopulmonary dysplasia?	5.535	6.423	7.222	**5.816**
115	Does targeted screening and needs assessment of social determinants (housing; safety; mental health; parental health literacy; transport needs;	5.597	6.237	5.556	**5.790**

	food security; community support) improve survival; quality of life and adverse events?				
116	In extremely preterm babies < 28 weeks is the use of neurally adjusted ventilatory assist (NAVA) ventilation with extubation to non-invasive NAVA (NIV NAVA) superior to conventional ventilation with extubation to NIPPV/ CPAP with regards to extubation success and bronchopulmonary dysplasia?	5.593	6.333	6.333	**5.770**
117	In preterm babies does sling provision and education to facilitate skin-to-skin contact improve parental wellbeing and weight gain when compared to standard care?	5.276	6.590	6.200	**5.760**
118	In extremely preterm infants with established chronic lung disease at 36 weeks corrected age; does sildenafil treatment improve survival compared with a placebo?	5.748	5.696	6.000	**5.756**
119	In preterm babies requiring parenteral nutrition does high amino acid intake improve growth and long term metabolic outcomes compared to standard intake?	5.807	5.724	5.143	**5.750**
120	Does implementation of formal parent peer support with meetings; social media and one-to-one peer support improve length of stay; readmissions and parental mental health compared to informal parental peer support?	5.289	6.500	6.300	**5.742**
121	In ex-preterm babies discharged home; does a community based sensory processing workshop improve infant regulation; improve bonding and decrease autism spectrum disorders compared to routine care?	5.342	6.378	6.444	**5.738**
122	For subsequent pregnancies after a neonatal admission; would a dedicated antenatal counselling and support programme improved parental anxiety and neonatal outcomes compared to no support programme?	5.163	6.447	7.600	**5.734**
123	In all infants receiving invasive or non-invasive respiratory support does prone or supine positioning improve the number of ventilation days and duration of supplemental oxygen?	5.327	6.067	8.000	**5.690**
124	Does an occupational therapist led parental training session and ongoing support regarding reading infant cues and participation in caregiving improve bonding and parental confidence compared to standard parental support?	5.039	6.667	6.778	**5.672**

125	In preterm infants at risk of necrotising enterocolitis does treatment with gut-derived IFN-y-releasing CD4+ T cells improve survival; brain injury and quality of life when compared to current practice?	5.403	6.875	6.833	**5.671**
126	In babies requiring long term parenteral nutrition (PN); does supporting an earlier discharge home (when stable PN established) compared to standard care improve quality of life for families without increased risk?	5.333	6.147	7.125	**5.667**
127	In term infants with congenital diaphragmatic hernia is extracorporeal membrane oxygenation (ECMO) or high-frequency oscillatory ventilation (HFOV) superior to improve survival; brain injury and quality of life?	5.358	6.360	7.125	**5.664**
128	In preterm babies requiring parenteral nutrition does high vitamin D intake improve growth and long-term metabolic outcomes compared to standard intake?	5.678	5.655	5.375	**5.653**
129	In formula fed babies born moderately preterm at 34 to 37 weeks gestation does enhanced nutrition support improve growth and long term cognition; when compared to term formula?	5.547	5.821	6.000	**5.650**
130	Do regular occupational therapy interventions for all babies admitted to NICU improve cognition; adverse events and decrease sensory processing disorders in comparison to minimal (<1 day per week) occupational therapy input?	5.218	6.275	6.556	**5.646**
131	In all babies does next generation whole genome sequencing improve the diagnostic/prognostic yield compared to current newborn screening practices without adverse ethical issues?	5.622	5.333	6.625	**5.640**
132	Does an occupational therapist led staff training session about sensitive neonatal handling improve long term outcomes such as sensory processing disorders and improve staff-parent relationships; compared to standard staff training?	5.053	6.590	6.200	**5.629**
133	Does circadian rhythm entrainment with nocturnal melatonin improve survival and neurodevelopmental impairment in infants at high risk for neurological injury (preterm < 28 weeks with peri-ventricular leukomalacia/ grade 3/4 intra-ventricular haemorrhage or term babies with hypoxic ischaemic encephalopathy) when compared to placebo?	5.506	5.739	6.571	**5.624**

134	In ex-preterm infants is early or late weaning on solids preferable with regards to growth; feeding behaviours and allergy?	5.284	6.079	6.500	**5.580**
135	In all babies requiring pre-medications for intubation is propofol or fentanyl / suxamethonium superior in improving survival and bronchopulmonary dysplasia?	5.412	5.720	6.900	**5.577**
136	In extremely preterm babies in the delivery room; does oxygen targeting based on near infrared spectroscopy (NIRS) (rather than SpO2) lead to improved survival and other core neonatal outcomes?	5.265	6.320	7.167	**5.558**
137	For parents of preterm infants does detailed information about future health risks (stroke; high blood pressure and heart disease) improve quality of life; parental satisfaction and parental mental health compared to no information?	5.193	6.115	7.714	**5.552**
138	Does provision of regular neonatal occupational therapy improve parental and staff perceptions of the developmental benefits for high-risk infants; compared to minimal occupational therapy input?	5.013	6.325	6.500	**5.543**
139	In preterm babies requiring parenteral nutrition do lower energy:protein ratios improve growth and long term metabolic outcomes compared to standard ratios?	5.570	5.552	5.143	**5.541**
140	In babies born as part of multiple births what is the effect of separation at any part of the neonatal journey on cognition; quality of life and adverse events?	4.671	6.718	7.778	**5.540**
141	When considering stopping antibiotics on the NNU; does the use of procalcitonin reduce the duration of antibiotics courses without adverse events compared to standard management using C-reactive protein?	5.647	5.067	5.250	**5.537**
142	In all babies being treated for sepsis do continuous vancomycin infusions increase survival and sepsis when compared to traditional multiple daily dose regimens?	5.463	5.400	6.667	**5.525**
143	Is plasmalyte or sodium chloride a better choice of bolus fluid for preterm infants with presumed hypovolaemia or poor perfusion?	5.412	6.200	5.400	**5.524**
144	Does a necrotising enterocolitis scoring tool incorporating parental views improve survival; sepsis and necrotising enterocolitis compared to necrotising enterocolitis scoring tools incorporating only professional views?	5.092	6.682	6.375	**5.519**

145	In preterm infants does the use of freshly expressed maternal milk a couple of times a day (as opposed to standard care with frozen or refrigerated maternal milk) improve growth and long term neonatal outcomes?	5.351	5.561	6.600	**5.497**
146	In all preterm babies < 32 weeks does nasal intubation rather than oral intubation improve feeding problems at 3 months corrected age?	5.010	6.545	6.800	**5.479**
147	Does parental presence during handovers improve parental satisfaction without increasing handover duration; compared to parents being asked to step outside?	4.831	6.282	7.300	**5.476**
148	In infants requiring sedation during ventilation does dexmedetomidine improve cognition and quality of life compared to standard care with an opiate infusion?	5.263	6.000	6.143	**5.471**
149	In babies on the postnatal ward receiving IV antibiotics for risk factors with a raised C-reactive protein; does performing a lumbar puncture (compared to not performing a lumbar puncture) lead to prolonged duration of stay or any adverse events?	5.274	5.875	6.429	**5.470**
150	In babies transferred between different NICUs does a psychologist supported 'repatriation' training package for parents improve parental mental health?	4.909	6.436	6.000	**5.468**
151	In preterm babies requiring parenteral nutrition does high vitamin A intake improve growth and long-term metabolic outcomes compared to standard intake?	5.437	5.621	5.250	**5.468**
152	Does giving an extra dose of caffeine prior to planned extubation or within 2 hours of unplanned extubation increase extubation success (remaining extubated at 5 days) compared to standard caffeine therapy?	5.163	5.889	6.833	**5.441**
153	In extremely preterm infants does 30 minutes of daily conversation with a parent or caregiver improve cognition and quality of life compared with no dedicated conversation?	4.865	6.154	6.778	**5.418**
154	In infants at risk of hypoglycaemia on the postnatal ward; does the use of donor milk for feed supplementation (as opposed to standard care with formula or glucose gel) improve maternal breast-feeding rates; hypoglycaemia episodes and quality of life?	5.053	5.821	7.333	**5.406**

155	Do tongue-tie interventions (laser or ligation) improve time taken to establish breast feeding; duration of breast feeding and parental satisfaction?	5.242	5.605	6.100	**5.399**
156	In preterm infants do routine clotting screens on admission (with corrections of derangements) lead to improve survival; brain injury and cognition compared to not performing a clotting screen unless clinically indicated?	5.361	5.176	6.286	**5.393**
157	In preterm babies does enteral insulin administration improve survival; sepsis and necrotising enterocolitis when compared to a masked placebo medication?	5.494	4.769	6.125	**5.380**
158	In babies with direct antiglobulin test (DAT) positive jaundice on the postnatal ward; does routine folic acid supplementation improve cognition; quality of life and adverse events?	5.262	5.600	5.875	**5.366**
159	In 28 to 32 week infants on non-invasive respiratory support is the best method to determine the need for surfactant administration lung ultrasound scoring or clinical signs/X-ray?	5.257	5.560	5.900	**5.360**
160	In preterm babies requiring parenteral nutrition does high folic acid intake improved growth and long-term metabolic outcomes compared to standard intake?	5.291	5.552	5.250	**5.350**
161	Would implementation of a ‘buddy system’ during the immediate postnatal period between neonatal and maternity staff looking after admitted babies and admitted mothers help to ensure parental presence at key neonatal aspects alongside managing maternal health; thus preventing early discharge and improving breast-milk production?	4.842	6.105	6.111	**5.325**
162	In preterm infants with suspected necrotising enterocolitis undergoing laparotomy does tranexamic acid improve survival and adverse events compared to no tranexamic acid treatment?	5.118	5.750	6.333	**5.296**
163	In preterm infants; does delivery and management in a ‘newborn individualized developmental care and assessment programme (NIDCAP) certified unit improve cognition; length of stay and breast-feeding rates when compared to management in a non-NIDCAP certified unit?	4.592	6.867	5.833	**5.268**
164	Does zinc supplementation of preterm infants improve growth?	5.266	5.032	5,889	**5.254**

165	In infants undergoing surgery does routine cerebral near infrared spectroscopy (NIRS) monitoring to target optimal analgesia improve cognition when compared to routine care?	5.125	5.500	5.857	**5.243**
166	In infants post-surgery does oxycodone analgesia reduce length of ventilation and length of hospital stay when compared to standard care with an opiate infusion?	5.039	5.762	6.000	**5.243**
167	How can 'memory-milk-gift-initiatives' best be implemented to support bereaved mothers with donating breast milk; and do these initiatives improve parental experiences and maternal mental health?	4.852	5.675	6.900	**5.239**
168	In infants who have been successfully intubated is ultrasound or X-ray the best method to confirm endotracheal tube tip position and avoid adverse events?	5.118	5.240	6.100	**5.212**
169	In babies diagnosed with brain injury; does specialist neonatal music therapy improve cognition; quality of life and short term physiological parameters when compared with standard neonatal care?	4.766	5.838	6.300	**5.210**
170	In preterm infants on the neonatal unit requiring respiratory support (invasive or non-invasive) does respiratory syncytial virus (RSV) prophylaxis improve survival and bronchopulmonary dysplasia compared to standard care (no RSV prophylaxis whilst an inpatient)?	4.842	5.480	7.778	**5.156**
171	Does a dedicated sibling support pack improve neonatal outcomes and family bonding; compared to no sibling support pack?	4.481	6.237	5.818	**5.127**
172	Do individualised infant diaries improve parental mental health and family bonding compared to no infant diary?	4.675	5.872	5.667	**5.120**
173	In clinically well term babies with > 10% weight loss does performing serum electrolyte measurements decrease survival; sepsis and seizures when compared to not measuring electrolytes?	4.893	5.720	5.571	**5.112**
174	In clinically well babies noted to have a raised cord lactate (but no TOBY criteria met) does observation and detailed clinical assessment lead to reduced length of stay and increased breast-feeding rates when compared to sequential blood gases with possible admission for intravenous fluids?	5.024	5.474	5.000	**5.103**

175	In intubated preterm infants does a short course of diuretics prior to extubation improve bronchopulmonary dysplasia and cognition when compared to a placebo?	4.901	5.400	5.778	**5.052**
176	In term babies establishing breast-feeding on the post-natal ward; does the use of cup; syringe or spoon feeding increase the aspiration risk over bottles?	4.872	5.308	5.700	**5.049**
177	In infants born to mothers with a history of maternal thyrotoxicosis; does inpatient observations for 48 hours with follow-up thyroid blood tests on day 5 lead to improved survival; cognition and adverse events when compared to routine postnatal care with detailed safety net advice?	5.123	4.941	4.333	**5.048**
178	In preterm babies with hyperbilirubinaemia; does using specific phototherapy radiance improve survival; brain injury and cognition when compared to using maximal phototherapy treatment?	4.707	5.900	6.143	**5.018**
179	In babies receiving end of life care; does specialist neonatal music therapy improve quality of life; parental experience and bereavement support in comparison to standard end of life care?	4.526	5.784	5.700	**5.000**
180	In neonates requiring surgery does deferring surgery until bilirubin is below a certain clinical level improve wound healing; length of stay and readmission rates?	4.743	5.784	5.700	**4.989**
181	In unwell term infants admitted to the NICU; does the routine addition of anti-viral treatments improve survival and quality of life compared to standard treatment with antibiotics only?	4.524	5.368	7.100	**4.894**
182	In preterm babies does use of a fluidised positioning pillow from birth improve incidence of scaphocephaly or plagiocephaly at discharge?	4.378	6.200	5.333	**4.890**
183	In preterm infants with patent ductus arteriosus does treatment with indomethacin improve survival when compared to placebo treatment?	4.682	5.647	5.167	**4.861**
184	In neonates post-surgery does giving prophylactic antibiotics only if there is a left shift on the differential white blood cell count improve survival; sepsis and adverse events compared with no antibiotics?	4.354	5.188	5.500	**4.554**
185	Does routinely sending the endotracheal tube tip for microscopy, culture and sensitivity after extubation increase survival and sepsis?	3.904	4.500	5.857	**4.130**

186	In preterm babies does taking a bath on the neonatal unit prior to discharge increase the risk of respiratory infections or other adverse events; when compared with no bath?	3.623	4.568	4.778	**3.992**
